# Therapeutic Effect of Melittin–dKLA Targeting Tumor-Associated Macrophages in Melanoma

**DOI:** 10.3390/ijms23063094

**Published:** 2022-03-13

**Authors:** Ik-Hwan Han, Chanmi Jeong, Juwon Yang, Seung-Hyeok Park, Deok-Sang Hwang, Hyunsu Bae

**Affiliations:** 1 Department of Physiology, College of Korean Medicine, Kyung Hee University, 26 Kyungheedae-ro, Dongdaemoon-gu, Seoul 02447, Korea; hihan@khu.ac.kr (I.-H.H.); wjdcksal34@naver.com (C.J.); evolymeb@gmail.com (J.Y.); 2 Department of Science in Korean Medicine, College of Korean Medicine, Kyung Hee University, 26 Kyungheedae-ro, Dongdaemoon-gu, Seoul 02447, Korea; 3 Department of Clinical Korean Medicine, Graduate School, Kyung Hee University, 26 Kyungheedae-ro, Dongdaemoon-gu, Seoul 02447, Korea; baomose@khu.ac.kr

**Keywords:** melanoma, melittin–dKLA, tumor-associated macrophage, M2 macrophage, therapeutic agent

## Abstract

Melanoma is an immunogenic tumor and a serious type of skin cancer. Tumor-associated macrophages (TAMs) express an M2-like phenotype and are involved in all stages of melanomagenesis; it is hence a promising target for cancer immunotherapy. We herein investigated whether melittin–dKLA inhibits the growth of melanoma by inducing apoptosis of M2-like macrophages. For the in vitro study, a conditioned medium of macrophages was prepared from M0, M1, or M2-differentiated THP-1 cells with and without melittin–dKLA. The affinity of melittin for M2 macrophages was studied with FITC (fluorescein isothiocyanate)-conjugated melittin. For the in vivo study, murine melanoma cells were inoculated subcutaneously in the right flank of mice, melittin–dKLA was intraperitoneally injected at 200 nmol/kg every three days, and flow cytometry analysis of TAMs was performed. Since melittin binds preferentially to M2-like macrophages, melittin–dKLA induced more caspase 3 expression and cell death in M2 macrophages compared with M0 and M1 macrophages and melanoma cells. Melittin–dKLA significantly inhibited the proliferation and migration of M2 macrophages, resulting in a decrease in melanoma tumor growth in vivo. The CD206^+^ M2-like TAMs were reduced, while the CD86^+^ M1-like TAMs were not affected. Melittin–dKLA is therapeutically effective against melanoma by inducing the apoptosis of M2-like TAMs.

## 1. Introduction

Melanoma develops in the pigment-producing cells and is the most serious among the types of skin cancer, which also include basal cell carcinoma (BCC) and squamous cell carcinoma (SCC) [1]. The worldwide incidence of melanoma has been increasing rapidly over the last 50 years, especially in the fair-skinned populations [2,3]. Interestingly, melanoma has been found to occur 1.5 times more frequently in men than in women; however, the prevalence before age 40 is higher in women than in men [4,5]. Although treatments such as surgery, radiotherapy, and chemotherapy are successful, many patients with melanoma still die from distal metastasis because of the aggressive nature of this cancer [6]. In particular, metastatic melanoma occurs in about 20% of the patients and can spread to other parts of the body, such as the lymph nodes and breasts [7]. Furthermore, the formation of metastasis is regulated by the tumor microenvironment (TME), which supports tumor progression and invasiveness [8]. The TME of melanoma is known to contain various types of cells, including endothelial cells, infiltrating immune cells, and cancer-associated fibroblasts (CAFs) [9,10]. Among these immune cells, tumor-associated macrophages (TAMs) are the major constituent of the melanoma microenvironment and are associated with poor prognosis [11]. Therefore, melanoma has been identified as one of the immunogenic tumors [12].

Macrophages (the so-called TAMs) are the most predominant immune cells in the tumor microenvironment and play a critical role in tumor pathogenesis [13]. TAMs are primarily made up of M2-like macrophages and play a key role in growth, survival, angiogenesis, and metastasis in the tumor microenvironment [14]. Moreover, they have been recognized as essential participants in the growth and survival of cutaneous melanoma [15]. Thus, M2-like TAMs could be an important target in anticancer therapy for melanoma.

Immune checkpoint inhibitors (ICIs) targeting the dysfunctional immune system to induce the tumor cells killing by CD8^+^ T cells have been used for the treatment of a variety of metastatic solid tumors, including melanoma [16]. Among immune checkpoint inhibitors, cytotoxic T-lymphocyte-associated protein-4 (anti-CTLA4; ipilimumab) and programmed cell death protein-1 (anti-PD-1; pembrolizumab, nivolumab) antibodies have revolutionized the management of advanced melanoma by showing tumor regression in nearly 50% of patients, compared to less than 10% in the past [17]. Unfortunately, data accumulated in recent years suggest that 40–60% of patients do not achieve a meaningful therapeutic effect, and a significant proportion of patients experience tumor recurrence within 2 years [18]. Furthermore, recent studies have reported that TAMs are involved in the failure of conventional chemotherapy as well as the failure of the anti-tumor immune surveillance and immunotherapy using ICIs [16]. 

Melittin (MEL), a major component of honey bee venom, consists of 26 amino acid residues [19]. It has been reported to have membrane-perturbing effects, including pore formation, fusion, and vesiculation, and therefore has non-specific cytotoxicity [20,21,22]. In a previous study, a new synthetic peptide was designed using the GGGGS linker between melittin and peptide d(KLAKLAK)2 (KLA), which modified the all-d enantiomer form to avoid degradation by proteases; this peptide demonstrated anticancer effects in a lung cancer model by inducing the apoptosis of M2-like TAMs [23].

The aim of this study was to determine whether melittin–dKLA selectively induces apoptosis in M2 macrophages and inhibits tumor growth in melanoma. Thus, these findings could provide a novel strategy to employ therapeutic agents, which target M2-like TAMs in melanoma.

## 2. Results

### 2.1. Differentiation of M2 Phenotype in THP-1-Derived Macrophages

**Figure 1:**
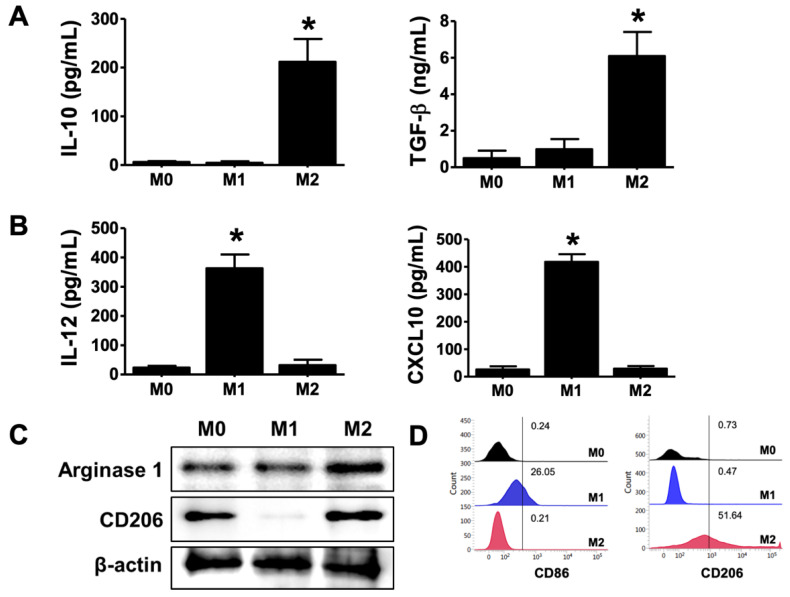
Differentiation of M2 phenotype in THP-1-derived macrophages. Human monocytes (THP-1) were treated with 100 nM PMA for 24 h and cultured with the following: 100 ng/mL LPS and 20 ng/mL rhIFN-γ for M1 macrophage differentiation; 20 ng/mL rhIL-4 and 20 ng/mL rhIL-13 for M2 macrophage differentiation. (**A**) Production of IL-10 and TGF-β as M2 macrophage markers was measured by ELISA. (**B**) Production of IL-12 and CXCL10 as M1 macrophage markers was measured by ELISA. (**C**) Expression of arginase 1 and CD206 protein was determined by Western blot as M2 macrophage markers. (**D**) Expression of CD86 and CD206 was measured by flow cytometry. All data are presented as means ± SD; * *p* < 0.05 versus the M0.

To test differentiation of macrophages to M2 phenotype, the THP-1 cells were stimulated with LPS and IFN-γ for M1 and with IL-4 and IL-13 for M2, and the polarization of macrophages was determined through the expression of M2 macrophage markers, such as IL-10, TGF-β, arginase-1, and CD206, and M1 macrophage markers, such as IL-12 and CXCL10. As shown in Figure 1A–D, M2 macrophages increased the production of IL-10 and TGF-β and increased the expression of arginase 1 and CD206. However, M1 macrophages showed increased production of IL-12 and CXCL10 and increased expression of CD86. Thus, these differentiated cells were used in assays to determine the effect of melittin–dKLA on M2 macrophages.

### 2.2. Affinity of Melittin in THP-1-Derived M2 Macrophages

**Figure 2:**
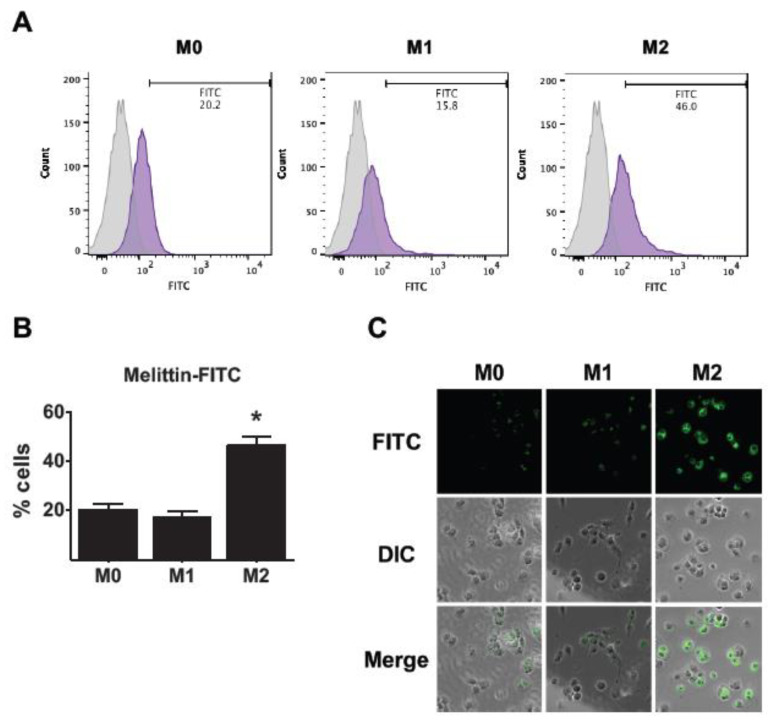
Affinity of melittin in THP-1-derived M2 macrophages. Human monocytes (THP-1) were treated with 100 nM PMA for 24 h and cultured with the following: 100 ng/mL LPS and 20 ng/mL rhIFN-γ for M1 macrophage differentiation; 20 ng/mL rhIL-4 and 20 ng/mL rhIL-13 for M2 macrophage differentiation. Cells were treated with melittin conjugated FITC (100 nM) at 37 °C for 1 h. (**A**) Affinity of Melittin in macrophages was measured by flow cytometry. (**B**) Representative graph generated from FACs analysis showing the percentage of FITC-positive cells. (**C**) Melittin–FITC bound to macrophages was determined by fluorescence microscopy. All data are presented as means ± SD; * *p* < 0.05 versus the M0.

To test whether melittin binds selectively to M2 macrophages, FITC-conjugated melittin was used, and the percentage of FITC-positive cells was measured with flow cytometry. Melittin was shown to bind to M0, M1, and M2 macrophages at 21%, 17%, and 46%, respectively (Figure 2A,B). It was seen that melittin had a higher affinity for M2 macrophages than M0 and M1 macrophages. In addition, fluorescence microscopy showed that melittin binds more to M2 macrophages compared with M0 and M1 macrophages (Figure 2C). These results suggest that melittin binds preferentially to M2 macrophages.

### 2.3. Apoptosis of THP-1-Derived M2 Macrophages by Melittin–dKLA

**Figure 3:**
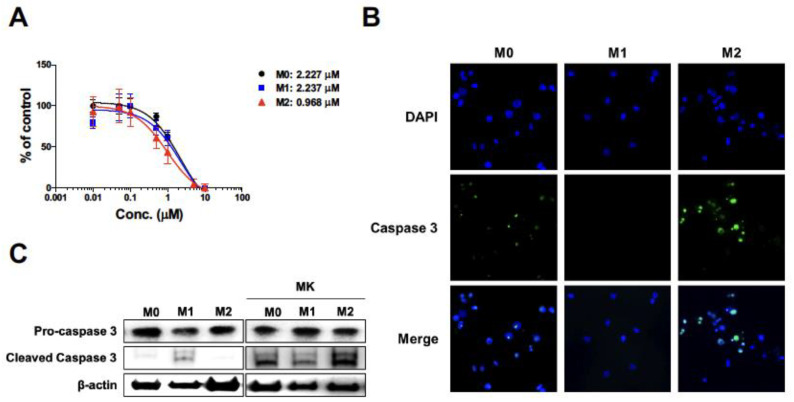
Apoptosis of THP-1-derived M2 macrophages by melittin–dKLA. Human monocytes (THP-1) were treated with 100 nM PMA for 24 h and cultured with the following: 100 ng/mL LPS and 20 ng/mL rhIFN-γ for M1 macrophage differentiation; 20 ng/mL rhIL-4 and 20 ng/mL rhIL-13 for M2 macrophage differentiation. (**A**) Macrophages were treated with melittin–dKLA by concentrations from 0.01 μM to 10 μM. Cell viability of macrophages was measured by CCK-8 assay. Data are presented as the means ± SD. (**B**,**C**) Macrophages were treated with melittin–dKLA (1 μM). Expression of caspase 3 was observed by fluorescence microscopy (**B**) and Western blot (**C**), MK: melittin–dKLA.

To determine the induction of M2 macrophage apoptosis by melittin–dKLA, the THP-1-derived macrophages were treated with melittin–dKLA at concentrations ranging between 0.01 and 10 μM after differentiation into M0, M1, and M2; the cell viability was then measured using the CCK-8 assay. The half-maximal (50%) inhibitory concentration (IC50) of melittin–dKLA in macrophages was evaluated as 2.227, 2.237, and 0.968 μM in M0, M1, and M2, respectively. Thus, melittin–dKLA induced cell death at a lower concentration in M2 macrophages than in other macrophages (Figure 3A). Moreover, the expression of caspase 3 related to cell apoptosis in macrophages treated with melittin–dKLA was determined by fluorescence microscopy and Western blotting. M2 macrophages showed increased expression of cleaved caspase 3 compared with M0 and M1 macrophages (Figure 3B,C). These results indicate that melittin–dKLA selectively induces the apoptosis of M2 macrophages.

### 2.4. Cytotoxicity of Melittin–dKLA in THP-1-Derived M2 Macrophages and SK-MEL-28 Cells

**Figure 4:**
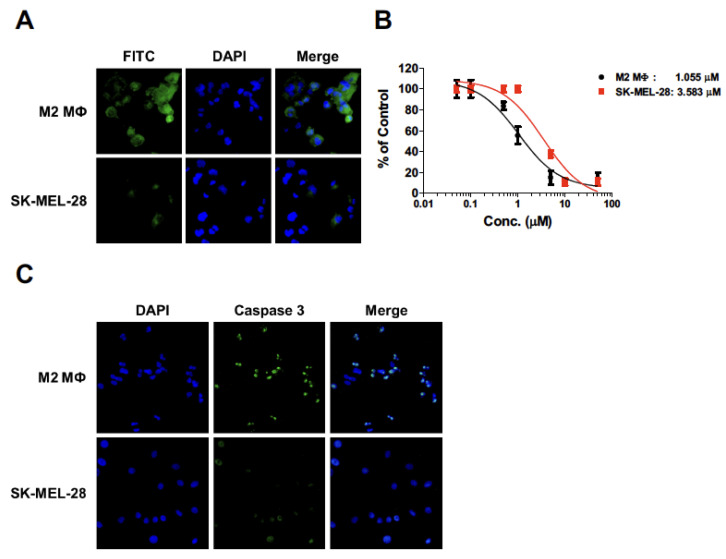
Cytotoxicity of melittin–dKLA in THP-1-derived M2 macrophages and SK-MEL-28 cells. Human monocytes (THP-1) were treated with 100 nM PMA for 24 h and cultured with the following: 20 ng/mL rhIL-4 and 20 ng/mL rhIL-13 for M2 macrophage differentiation. (**A**) M2 macrophages and SK-MEL-28 cells were treated with melittin conjugated FITC (100 nM). Melittin–FITC bound to cells was determined by fluorescence microscopy. (**B**) M2 macrophages and SK-MEL-28 cells were treated with melittin–dKLA by concentrations from 0.05 μM to 50 μM. Cell viability was measured by CCK-8 assay. Data are presented as the means ± SD. (**C**) Cells were treated with melittin–dKLA (1 μM). Expression of caspase 3 was observed by fluorescence microscopy.

To test whether melittin–dKLA selectively affects M2 macrophages, the affinity of melittin–FITC and the cytotoxicity of melittin–dKLA were determined in M2 macrophages and melanoma cells. Melittin was shown to bind preferentially to THP-1-derived M2 macrophages compared with SK-MEL-28 cells (Figure 4A). Further, cell death by melittin–dKLA was induced more in M2 macrophages than in melanoma cells. The IC50 of melittin–dKLA was evaluated as 1.055 and 3.583 μM in M2 macrophages and SK-MEL-28 cells, respectively (Figure 4B). Moreover, the expression level of caspase 3 was higher in M2 macrophages than in SK-MEL-28 cells (Figure 4C). Thus, these results show that melittin–dKLA selectively induced apoptosis in M2 macrophages.

### 2.5. Proliferation and Migration in SK-MEL-28 Cells by Conditioned Medium of THP-1-Derived M2 Macrophages Pretreated with Melittin–dKLA

**Figure 5:**
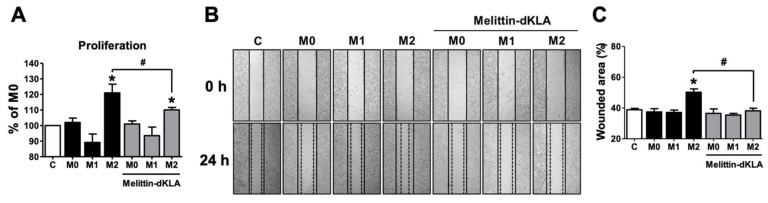
Proliferation and migration in SK-MEL-28 cells by conditioned medium of THP-1-derived M2 macrophages pretreated with melittin–dKLA. Human monocytes (THP-1) were treated with 100 nM PMA for 24 h and cultured with the following: 100 ng/mL LPS and 20 ng/mL rhIFN-γ for M1 macrophage differentiation; 20 ng/mL rhIL-4 and 20 ng/mL rhIL-13 for M2 macrophage differentiation. The differentiated macrophages were pretreated with melittin–dKLA (1 μM) for 1 h. For preparation of macrophages, macrophages were incubated with serum-free media for 24 h and then supernatants were harvested by syringe filter (0.2 μm). (**A**) Proliferation of SK-MEL-28 cells was measured by CCK-8 assay. (**B**) Migration of SK-MEL-28 cells was determined by wound healing assay. (**C**) The percent of each wounded area was calculated as: [(mean wounded breadth − mean remaining breadth)]/mean wounded breadth × 100. The graph presents the percentage of wounded area. All data are presented as means ± SD; * *p* < 0.05 versus the Control (**C**), # *p* < 0.05 versus the M2.

M2 macrophages in the tumor microenvironment have been reported to be associated with tumor-promoting behaviors, including proliferation, invasion, and metastasis. As shown in Figure 3, melittin–dKLA induced selective apoptosis of M2 macrophages. We tested whether melittin–dKLA inhibits the proliferation and migration of melanoma cells by targeting M2 macrophages. First, the conditioned medium from M0, M1, and M2 macrophages was pretreated with melittin–dKLA, and the SK-MEL-28 cells were then treated with the conditioned medium. The proliferation of SK-MEL-28 cells was significantly increased in the conditioned medium of M2 macrophages compared with the conditioned media of M0 and M1 macrophages. However, the conditioned medium of M2 macrophages pretreated with melittin–dKLA significantly inhibited the proliferation of SK-MEL-28 compared with the conditioned medium of M2 macrophages, whereas the conditioned media of M0 and M1 macrophages pretreated with melittin–dKLA did not affect the proliferation of SK-MEL-28 (Figure 5A). Next, the migration of melanoma cells was measured using a wound-healing assay. As shown in Figure 5A, the migratory capability of SK-MEL-28 cells was also enhanced in the conditioned medium of M2 macrophages but was inhibited significantly in the conditioned medium of M2 macrophages pretreated with melittin–dKLA (Figure 5B,C). Thus, these findings suggest that melittin–dKLA inhibited the proliferation and migration of melanoma cells by targeting M2 macrophages.

### 2.6. Inhibition of Tumor Growth by Melittin–dKLA in Mouse Model of Melanoma

**Figure 6:**
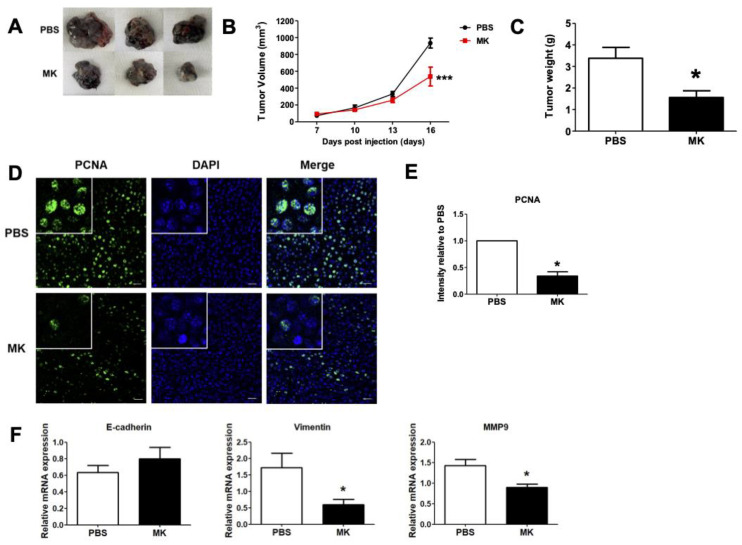
Inhibition of tumor growth by melittin–dKLA in a mouse model of melanoma. (**A**) Images show the tumor tissues from the mouse model. (**B**) The volume of the tumor was measured by a digital caliper and calculated using the formula V = (Width (2) × Length)/2. (**C**) The tumor weight of mice was measured by electronic scale after sacrifice. (**D**) Expression of PCNA in tumor tissues was determined by immunofluorescence staining assay. Confocal images of a tumor tissue slice stained for PCNA (green) and nucleus (DAPI; blue). (**E**) The graph is presented as intensity relative to PBS. (**F**) Expression of E-cadherin, vimentin, and MMP9 mRNA in tumor tissues was analyzed by quantitative real-time PCR. All data are presented as means ± SD (*n* = 5); * *p* < 0.05, *** *p* < 0.0001 versus the PBS group, MK: melittin–dKLA.

To determine the effect of melittin–dKLA in a mouse melanoma model, C57BL/6 mice were inoculated subcutaneously with B16F10 cells (1 × 10^6^/mouse) in the right flank (*n* = 5/group). Melittin–dKLA (200 nmol/kg) was intraperitoneally administered every 3 days (4 times) at 7 days after tumor inoculation. The measured tumor volume and weight indicated that the tumor volume was significantly decreased in the melittin–dKLA group compared with the PBS group (Figure 6A,B). Similarly, the tumor weight was reduced in the melittin–dKLA group (Figure 6C). Further, the melittin–dKLA group showed significant decreases in the expression of PCNA compared with the PBS group in tumor tissues (Figure 6D,E). Although the expression of E-cadherin, an epithelial marker, was not significantly increased by melittin–dKLA, the expression of vimentin and the MMP9 gene related to the epithelial–mesenchymal transition (EMT) was decreased in the melittin–dKLA group compared with the PBS group (Figure 6F). These results suggest that melittin–dKLA has an inhibitory effect on melanoma in vivo.

### 2.7. Reduction of M2-like TAMs by Melittin–dKLA in Mouse Model of Melanoma

**Figure 7:**
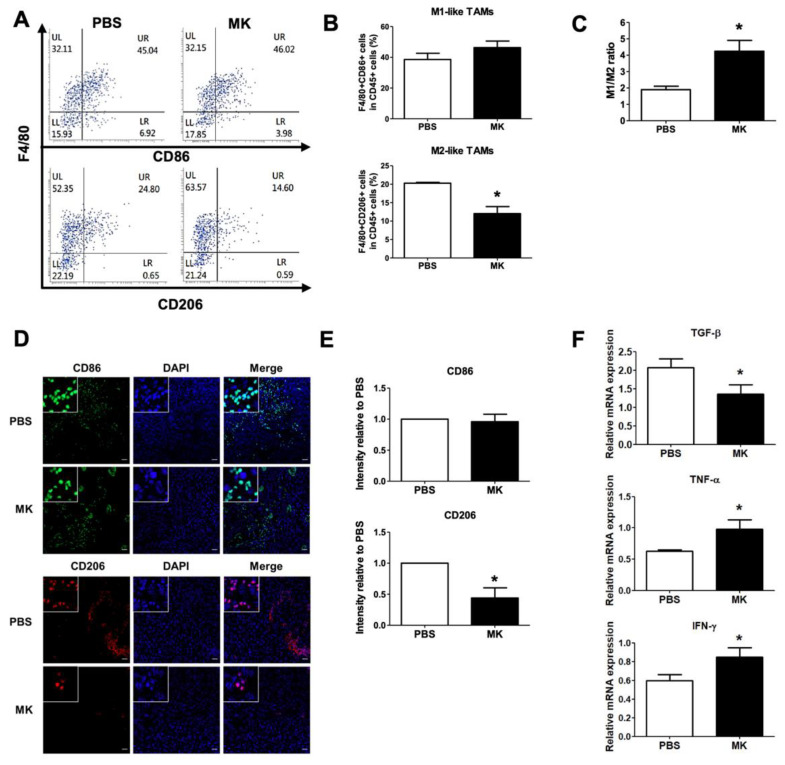
Reduction of M2-like TAMs by melittin–dKLA in a mouse model of melanoma. Single cells were harvested from tumor tissues. (**A**) M1-like TAMs were stained as CD45^+^F4/80^+^CD86^+^ (upper panel), and M2-like TAMs were marked as CD45^+^F4/80^+^CD206^+^ (bottom panel). The cells are shown as dot plots within the F4/80 and CD86 or CD206 axis gated on CD45^+^ cells. (**B**) Data for each cell phenotype are displayed as the percentages of F4/80^+^CD86^+^M1- (upper) and F4/80^+^CD206^+^ M2-like TAMs (bottom) in CD45^+^ cells. (**C**) The M1/M2 ratio was calculated on the basis of the percentage of F4/80^+^CD86^+^ M1-like and F4/80^+^CD206^+^ M2-like TAMs in CD45^+^ cells. (**D**) Expression of CD86 and CD206 in tumor tissues was determined by immunofluorescence staining assay. Confocal images of a tumor tissue slice stained for CD86 (green), CD206 (red), and nucleus (DAPI; blue). (**E**) The graph is presented as intensity relative to PBS. (**F**) Expression of TGF-β, TNF-α, and IFN-γ mRNA in tumor tissues was analyzed by quantitative real-time PCR. All data are presented as means ± SD (*n* = 5); * *p* < 0.05 versus the PBS group.

Melittin–dKLA showed a tendency to suppress the tumor growth of melanoma (Figure 6). To assess whether melittin–dKLA can reduce M2-like TAMs in tumor tissue, single cells from tumor tissues were harvested, and the numbers of F4/80^+^CD86^+^ M1 and F4/80^+^CD206^+^ M2-like TAMs in CD45^+^ leukocytes were measured by flow cytometry. The number of M2-like TAMs was significantly lower in the melittin–dKLA group than in the PBS group, while the number of M1-like TMAs showed no difference between the melittin–dKLA and PBS groups (Figure 7A,B). Interestingly, the M1/M2 ratio was significantly higher in the melittin–dKLA group than in the PBS group (Figure 7C). In tumor tissues, the melittin–dKLA group showed a decrease in CD206 positive cells compared with the PBS group (Figure 7D,E). Furthermore, the melittin–dKLA group showed a decrease in TGF-β mRNA expression and an increase in TNF-α and IFN-γ mRNA expression in tumor tissues (Figure 7F). These data indicate that melittin–dKLA induces suppression of tumor growth by reducing M2-like TAMs in a mouse melanoma model.

## 3. Discussion

Our study demonstrated that melittin–dKLA induces apoptosis by selectively binding to M2 macrophages and inhibits the growth of melanoma tumor cells.

Tumor-associated macrophages are the most common immune cells in the tumor microenvironment [24]. Macrophages are generally divided into two distinct types: M1 and M2 [25]. M1 macrophages have antitumor functions, while M2 macrophages have tumor-promoting functions [26]. M1 macrophages are polarized by lipopolysaccharides (LPS) and interferon (IFN)-γ and are characterized by the expression of IL-12, CXCL10, tumor necrosis factor (TNF)-α, and inducible nitric oxide synthase (iNOS) [27]. M2 macrophages are polarized by Th2 cytokines, such as IL-4 and IL-13, and are characterized by the expression of macrophage mannose receptor (MMR, also called CD206), arginase-1, and inflammatory cytokines, such as IL-10 and TGF-β [28]. In this study, we determined that CD86 expression and IL-12 and CXCL10 production occurred in macrophages treated with LPS and IFN-γ for M1 polarization, while CD206 and arginase-1 expression and IL-10 and TGF-β production were observed in macrophages treated with IL-4 and IL-13 for M2 polarization (Figure 1). The tumor microenvironment is predominantly composed of M2 macrophages, and their high density is closely associated with poor prognosis as well as mortality in various cancers [29,30,31]. Recently, many researchers have attempted to develop anticancer drugs targeting TAMs [32,33].

Melittin is the major component of honey bee venom, representing approximately 40–60% of the total composition [34]. It is widely used in the treatment of arthritis, frozen shoulder, ulcer, colitis, and cancer [35]. Melittin has been investigated for anticancer effects because of its ability to inhibit cell growth and induce apoptosis and necrosis of cells [36,37]. However, previous studies have reported that melittin binds preferentially to M2-like TAMs and has a suppressive effect on tumor progression in lung cancer [38]. Moreover, the peptide KLA, which is a naturally occurring antibacterial peptide, is a mitochondrial membrane-disrupting agent that binds to the negatively charged bacterial membrane. It is not bound to the eukaryotic plasma membrane and is not toxic to eukaryotic cells [39,40]. This peptide must be linked to other peptides to facilitate the cell-penetrating ability of KLA in eukaryotic cells [41,42].

In this study, we determined that melittin binds to human M2 macrophages with more affinity than M0 and M1 macrophages using FITC-conjugated melittin (Figure 2 and Figure 4). Furthermore, a previous study indicated that melittin–dKLA, with both melittin and the pro-apoptotic peptide dKLA, induced the release of cytochrome c by disrupting the mitochondrial membrane and selectively induced the apoptosis of M2 macrophages [23]. This study similarly showed that melittin–dKLA selectively induced apoptosis by expressing caspase 3 in M2 macrophages (Figure 3 and Figure 4).

Melanoma originating from the malignant transformation of melanocytes is the most aggressive form of skin cancer, and its incidence is increasing rapidly compared with other types of cancer [43,44]. In the early stages of metastasis, tumor cells acquire migratory and invasive properties [45]. Melanoma, which is an immunogenic tumor, is associated with immune cells residing in the TME [12]. Among these immune cells, M2 macrophages are known to be involved in tumor growth, angiogenesis, and metastasis in the TME [46]. In this study, melanoma cells showed increased proliferative and migratory properties in a conditioned medium of M2 macrophages, which were, however, decreased by melittin–dKLA (Figure 5). Additionally, melittin–dKLA suppressed the tumor volume and weight in a mouse melanoma model (Figure 6A–E) and reduced the expression of EMT markers associated with cancer cell metastasis, thereby showing that melittin–dKLA could inhibit the metastasis of melanoma (Figure 6F). However, there are insufficient data on whether metastasis is inhibited by melittin–dKLA in vivo. In a future study, it will be necessary to evaluate the metastasis inhibitory efficacy of melittin–dKLA using a metastatic melanoma mouse model.

Recently, M2 macrophages in tumor tissues were shown to play an important role as a contributing factor in the formation of an immunosuppressive TME [47]. Thus, the enhanced anticancer effects of combination therapy with M2-like TAM-targeting agents on immune checkpoints are repeatedly observed in various cancer models, including lung cancer, colon cancer, melanoma, and breast cancer [48,49]. In this study, the number of M2-like TAMs in tumor tissue of melanoma was decreased by melittin–dKLA, while the number of M1-like TAMs was not affected. Importantly, the M1/M2 ratio in tumor tissues was increased by melittin–dKLA (Figure 7); this is expected to have anticancer effects because the increased proportion of M1-like TAMs performs proinflammatory functions in the TME because of the apoptosis of M2-like TAMs by melittin–dKLA.

These findings suggest that melittin–dKLA induces the suppression of tumor growth by selectively targeting M2-like TAMs in melanoma. Thus, melittin–dKLA derived from bee venom appears to be a promising antitumor agent for melanoma.

## 4. Materials and Methods

### 4.1. Cells and Mice

C57BL/6 wild-type mice were purchased from DBL (Chungbuk, Korea). The animal procedures were approved by the University of Kyung Hee Institutional Animal Care and Usage Committee (KHUASP(SE)-20-5302). All animals were maintained in a pathogen-free environment on a 12 h light/dark cycle and had access to food and water. The murine melanoma cell line (B16F10) was cultured in Dulbecco’s Modified Eagle’s medium (DMEM; Welgene, Gyeongsan, Korea) supplemented with 10% fetal bovine serum (FBS; Welgene), 100 U/mL penicillin, and 100 μg/mL streptomycin (Gibco; Thermo Fisher Scientific Inc., Waltham, MA, USA). The human monocytic leukemia cell line (THP-1) was cultured in a medium (RPMI1640; Welgene) supplemented with 10% fetal bovine serum (FBS; Welgene), 100 U/mL penicillin, and 100 μg/mL streptomycin (Gibco). The cells were cultured every 2–3 days until the cells became 80% confluent, and they were then incubated at 37 °C with 95% humidity and 5% CO_2_ for all experiments.

### 4.2. Differentiation of Macrophages and Preparation of Conditioned Medium

THP-1 monocytes were treated with 100 nM phorbol 12-myristate 13-acetate (PMA; Sigma-Aldrich, St. Louis, MO, USA) for 24 h. The monocytes were treated with RPMI1640 supplemented with 5% FBS for 72 h to obtain differentiated non-polarized phenotype M0 cells. To generate polarized phenotypes, cells were incubated for 72 h with 20 ng/mL recombinant human interferon (rhIFN)-γ (Peprotech; Rocky Hill, NJ, USA) and with 100 ng/mL lipopolysaccharides (LPS; Sigma-Aldrich) to obtain M1 macrophages; the cells were incubated with 20 ng/mL recombinant human interleukin (rhIL)-4 (Peprotech) and 20 ng/mL rhIL-13 (Peprotech) to obtain M2 macrophages. To prepare a conditioned medium for THP-1-derived macrophages, the THP-1-derived macrophages that differentiated into M0, M1, and M2 macrophages were replaced with a serum-free medium to remove the cytokine-containing medium and were then incubated for 24 h. The supernatants of the cells were then collected and identified using 0.2 μm syringe filters (GVSm Sanford, ME, USA).

### 4.3. Tumor Inoculation and Animal Study

For tumor model generation, the B16F10 cells were mixed with Matrigel (Corning, NY, USA), and female C57BL/6 wild-type mice (6–8 weeks old) were inoculated subcutaneously with 1 × 106 cells per mouse in the right flank (*n* = 5/group). Melittin–dKLA (GenScript Corporation, Peace Catterway, NJ, USA) (200 nmol/kg) was administered every 3 days intraperitoneally, 7 days after tumor inoculation. Tumor size was examined with a digital caliper every 3 days, and tumor volume was calculated using the formula V = (Width (2) × Length)/2. According to the guidelines, mice were killed when the tumor size attained a maximum diameter of 1–1.5 cm after inoculation.

### 4.4. Tissue Cell Preparation and Flow Cytometry Analysis

Tumor tissues were chopped into thin pieces and separated in DNase I (1 U/mL) (Roche, Indianapolis, IN, USA) and collagenase D (1 mg/mL) (Roche) in serum-free RPMI1640 (Welgene) for 30 min at 37 °C with a shaking incubator. The tissues were gently dissociated using the MACS dissociator and the MACS C tube (Milteny Biotec, Auburn, CA, USA). The tissues were separated using a 100 μm nylon mesh strainer. The single cells were then passed through a 40 μm nylon mesh strainer. Red blood cells were eliminated with 1X RBC lysing buffer, single cells were washed, and antibodies were stained. The following antibodies were purchased from BD Bioscience: mouse CD45-FITC, F4/80-PE, CD86-PE-CY7, and CD206-APC. The cells were assessed for the percentage of double-positive cells of F4/80 and CD86 for M1 or F4/80 and CD206 for M2 macrophages in CD45 cells. The M1/M2 ratio was calculated as the percentage of each double-positive for M1 and M2 macrophages.

To identify the differentiation of THP-1-derived macrophages, the THP-1 cells were differentiated into M0, M1, or M2 macrophages by the method described above. The cells were washed, and the antibodies were stained. The following antibodies were purchased from BD Bioscience: human CD86-FITC and CD206-APC. 

To test the affinity of melittin for M2 macrophages, the THP-1-derived macrophages that were differentiated into M0, M1, and M2 macrophages were incubated with 100 nM melittin–FITC (GenScript Corporation, Peace Catterway, NJ, USA) for 1 h at 37 °C. All data were detected with a FACSLyric System (BD Bioscience, CA, USA) and analyzed with FlowJo software (BD Bioscience, OR, USA).

### 4.5. Immunofluorescence Staining

THP-1 cells were seeded at 5 × 10^4^ cells/well on cover glass (NUNC; Thermo Fisher Scientific Inc., Waltham, MA, USA) in a 24-well plate. The cells were differentiated into M0, M1, and M2 macrophages by the aforementioned method and then treated with melittin–dKLA (1 μM) for 24 h. The cells were washed, fixed with 4% paraformaldehyde for 10 min at −20 °C, and blocked with 0.1% normal goat serum for 1 h. The cover glasses were then incubated with anti-cleaved caspase 3 antibody (1:200, rabbit polyclonal) (Cell Signaling Technology, Danvers, MA, USA) overnight at 4 °C and then washed and stained with Alexa 488-conjugated anti-rabbit IgG secondary antibody (1:500) (Invitrogen, CA, USA) at 37 °C for 1 h. The cover glasses were mounted in Vectashield mounting medium (Vector Laboratories, Burlingame, CA, USA) with 4′,6-diamidino-2-phenylindole (DAPI) to visualize the nuclei. All images were photographed using the ZEISS LSM 800 laser scanning microscope (Bio-Rad, Richmond, CA, USA).

Tumor tissues were fixed overnight in 10% neutral buffered formalin and cut to 4 μm with a regular thickness after being embedded in paraffin. The sections were dipped in xylene and then 100%, 90%, 80%, and 70% ethanol, respectively, and washed in running tap water for rehydration. For melanin bleaching, the slides were immersed in 10% H_2_O_2_ solution diluted in PBS in a glass jar and placed in a pre-heated 65 °C dry oven. The tissue sections were washed in deionized water for 10 min after the bleaching procedure. The tissue antigen was heat-retrieved with sodium citrate buffer (pH 6.0) for 1 min at 121 °C. The tissue was incubated with 3% H_2_O_2_ for 15 min and blocked with 1.5% BSA containing 0.2% Triton X-100 for 1 h. The tissues were incubated with rat anti-mouse CD206, CD86, and rabbit anti-mouse PCNA primary antibodies (1:1000; Abd Serotec, Oxford, UK) and visualized using Alexa-488 conjugated anti-rat IgG and Alexa-594 conjugated anti-rabbit IgG (1:1000; Invitrogen, CA, USA). All antibodies were diluted in 0.5% BSA solution. Five random fields of different tumor nodules in the lung were detected with laser scanning confocal microscopy (Carl Zeiss, Jena, Germany).

### 4.6. ELISA

To assess the phenotypic changes in THP-1-derived macrophages, the THP-1 cells were differentiated into M0, M1, or M2 macrophages using the methods described above. The supernatants of the cells were harvested and stored at −20 °C. To quantify interleukin (IL)-10, IL-12, CXCL10, and tumor growth factor (TGF)-β levels, the supernatants were measured using BD OptEIA ELISA kits (BD Biosciences Inc., San Diego, CA, USA) according to the manufacturer’s instructions.

### 4.7. CCK-8 Assay

To examine the cytotoxic effect of melittin–dKLA in THP-1-derived macrophages, the THP-1 cells were seeded at 2 × 10^4^ cells/well in 96-well plates and differentiated into M0, M1, or M2 macrophages using the aforementioned methods. After the differentiation of macrophages, the cells were pretreated with melittin–dKLA (10, 5, 1, 0.5, 0.1, 0.05, and 0.01 μM) for 1 h and cultured in a serum-free medium for 24 h. For the proliferation of melanoma cells in the macrophage-conditioned medium, SK-MEL-28 cells were seeded at 0.5 × 10^4^ cells/well in 96-well plates and incubated with 20% conditioned medium of M0, M1, and M2 macrophages or M0, M1, and M2 macrophages pretreated with melittin–dKLA (1 μM) for 24 h. Cell death and proliferation were analyzed using the Cell Counting Kit-8 (CCK-8) assays. CCK-8 reagent (Enzo Life Sciences, Farmingdale, NY, USA) was added to each well at a ratio of 1/10 and the plates were incubated for 2 h. The absorbance was measured at 450 nm using a microplate reader (Molecular Devices, San Jose, CA, USA). Data are expressed as the means ± SD of three independent experiments.

### 4.8. Wound Healing Assay

The migration of melanoma cells was assessed using wound-healing assays. SK-MEL-28 cells were seeded in 24-well plates at 2 × 10^5^ cells/well and incubated with 10% FBS in RPMI1640. After reaching confluence, the cells were scratched by scraping the surface of the well with a sterile micropipette tip. The cells were cleaned immediately, and the wells were filled with serum-free medium or 20% conditioned media of macrophages and cultured for 24 h. Five or more different fields were photographed for the wound site of each sample before and after incubation using an inverted microscope (Olympus, Tokyo, Japan). The wound areas were measured using ImageJ software (NCI, Bethesda, MD, USA). The percentage of each wound site filled with cell migration was calculated as: [(mean wounded breadth − mean remaining breadth)/mean wounded breadth × 100]. The data are expressed as the means ± SD of three independent experiments.

### 4.9. Western Blot Analysis

THP-1 cells were differentiated into M0, M1, or M2 macrophages using the methods described above. The cells were obtained and lysed in PRO-PREP protein extraction solution (iNtRON, Bio Inc., Seongnam, Korea). The protein concentrations were measured with a Bradford protein assay reagent kit (Bio-Rad, Richmond, CA, USA). Proteins were separated using 10% SDS-polyacrylamide gel electrophoresis (PAGE) and transferred to polyvinylidene difluoride (PVDF) membranes. These were cultured as primary antibodies with anti-caspase 3, anti-cleaved caspase 3 (Cell Signaling Technology), anti-arginase 1, anti-CD206, and anti-β-actin Ab (1:1000) (Abcam). Goat anti-rabbit horseradish peroxidase-conjugated IgG or goat anti-mouse horseradish peroxidase-conjugated IgG (Abcam, Cambridge, MA, USA) were used as secondary antibodies. Protein bands were detected using a chemiluminescence reagent kit (SurModics, MN, USA).

### 4.10. Quantitative Real-Time PCR

Total RNA was extracted from lung tissues using an easy-BLUE RNA extraction kit (iNtRON Biotechnology, Korea). The cDNA synthesis was performed using cyclescript reverse transcriptase (Bioneer, Korea) following the manufacturer’s instructions. Quantitative real-time PCR was performed using the SensiFAST SYBR no-Rox kit (Bioline, Korea). The cDNA synthesis conditions were as follows: cycling conditions were 95 °C for 15 s, 55 °C for 10 s, and 72 °C for 10 s. Each reaction was performed in triplicate. The base sequences of the primers used were the following: TGF-β: forward, 5-CCA CCT GCA AGA CCA TCG AC-3; reverse, 5-CTG GCG AGC CTT AGT TTG GAC-3. E-cadherin: forward, 5-CAA GGA CAG CCT TCT TTT CG-3; reverse, 5-TGG ACT TCA GCG TCA CTT TG-3. Vimentin: forward, 5-TGA AGG AAG AGA TGG CTC GT-3; reverse, 5-TCC AGC AGC TTC CTG TAG GT-3. MMP9: forward, 5-TGA ATC AGC TGG CTT TTG TG-3; reverse, 5-ACC TTC CAG TAG GGG CAA CT-3. TNF-α: forward, 5′-ACG GCA TGG ATC TCA AAG AC-3′; reverse, 5′-GTG GGT GAG GAG CAC GTA GT-3′. IFN-γ forward, 5′-TTT GAG GTC AAC AAC CCA CA-3′; reverse, 5′-CGC AAT CAC AGT CTT GGC TA-3′. GAPDH: forward, 5′-CCC AGA AGA CTG TGG ATG G-3′; reverse, 5′-CAC ATT GGG GGT AGG AAC AC-3′.

### 4.11. Statistics

All data are expressed as means ± standard deviation. Statistical significance was analyzed by one-way ANOVA followed by Tukey’s multiple comparison tests and the two-tailed Mann–Whitney U test using Prism 5.01 software. Differences with *p*-values of <0.05 were considered statistically significant.

## Data Availability

All data generated or analyzed during this study are included in this published article.

## References

[1] Wu C.-E., Esfandiari A., Ho Y.-H., Wang N., Mahdi A., Aptullahoglu E., Lovat P., Lunec J. (2018). Targeting negative regulation of p53 by MDM2 and WIP1 as a therapeutic strategy in cutaneous melanoma. Br. J. Cancer.

[2] Rigel D.S., Carucci J.A. (2000). Malignant melanoma: Prevention, early detection, and treatment in the 21st century. CA Cancer J. Clin..

[3] Caini S., Gandini S., Sera F., Raimondi S., Fargnoli M.C., Boniol M., Armstrong B.K. (2009). Meta-analysis of risk factors for cutaneous melanoma according to anatomical site and clinico-pathological variant. Eur. J. Cancer.

[4] Rastrelli M., Tropea S., Rossi C.R., Alaibac M. (2014). Melanoma: Epidemiology, risk factors, pathogenesis, diagnosis and classification. In Vivo.

[5] Paulson K.G., Gupta D., Kim T.S., Veatch J.R., Byrd D.R., Bhatia S., Wojcik K., Chapuis A.G., Thompson J.A., Madeleine M.M. (2020). Age-Specific Incidence of Melanoma in the United States. JAMA Dermatol..

[6] CChambers A.F., Groom A.C., Macdonald I.C. (2002). Dissemination and growth of cancer cells in metastatic sites. Nat. Cancer.

[7] Al Samaraee A., Khout H., Barakat T., Fasih T. (2012). Breast metastasis from a melanoma. Ochsner J..

[8] Quail D.F., Joyce J.A. (2013). Microenvironmental regulation of tumor progression and metastasis. Nat. Med..

[9] Hanahan D., Coussens L.M. (2012). Accessories to the crime: Functions of cells recruited to the tumor microenvironment. Cancer Cell.

[10] Kerkar S.P., Restifo N.P. (2012). Cellular Constituents of Immune Escape within the Tumor Microenvironment: Figure 1. Cancer Res..

[11] Falleni M., Savi F., Tosi D., Agape E., Cerri A., Moneghini L., Bulfamante G.P. (2017). M1 and M2 macrophages’ clinicopathological significance in cutaneous melanoma. Melanoma Res..

[12] Tucci M., Passarelli A., Mannavola F., Felici C., Stucci L.S., Cives M., Silvestris F. (2019). Immune System Evasion as Hallmark of Melanoma Progression: The Role of Dendritic Cells. Front. Oncol..

[13] Schoppmann S.F., Birner P., Stöckl J., Kalt R., Ullrich R., Caucig C., Kriehuber E., Nagy K., Alitalo K., Kerjaschki D. (2002). Tumor-Associated Macrophages Express Lymphatic Endothelial Growth Factors and Are Related to Peritumoral Lymphangiogenesis. Am. J. Pathol..

[14] Lissbrant I.F., Stattin P., Wikstrom P., Damber J.E., Egevad L., Bergh A. (2000). Tumor associated macrophages in human prostate cancer: Relation to clinicopathological variables and survival. Int. J. Oncol..

[15] Bardi G.T., Smith M.A., Hood J.L. (2018). Melanoma exosomes promote mixed M1 and M2 macrophage polarization. Cytokine.

[16] Ceci C., Atzori M.G., Lacal P.M., Graziani G. (2020). Targeting Tumor-Associated Macrophages to Increase the Efficacy of Immune Checkpoint Inhibitors: A Glimpse into Novel Therapeutic Approaches for Metastatic Melanoma. Cancers.

[17] Carlino M.S., Larkin J., Long G.V. (2021). Immune checkpoint inhibitors in melanoma. Lancet.

[18] IImbert C., Montfort A., Fraisse M., Marcheteau E., Gilhodes J., Martin E., Bertrand F., Marcellin M., Burlet-Schiltz O., Peredo A.G. (2020). Resistance of melanoma to immune checkpoint inhibitors is overcome by targeting the sphingosine kinase-1. Nat. Commun..

[19] Eisenberg D. (1984). Three-dimensional structure of membrane and surface proteins. Annu. Rev. Biochem..

[20] Dempsey C. (1990). The actions of melittin on membranes. Biochim. Biophys. Acta (BBA)—Rev. Biomembr..

[21] Ladokhin A., Selsted M., White S. (1997). Sizing membrane pores in lipid vesicles by leakage of co-encapsulated markers: Pore formation by melittin. Biophys. J..

[22] Lee M.-T., Hung W.-C., Chen F.-Y., Huang H.W. (2008). Mechanism and kinetics of pore formation in membranes by water-soluble amphipathic peptides. Proc. Natl. Acad. Sci. USA.

[23] Lee C., Jeong H., Bae Y., Shin K., Kang S., Kim H., Oh J., Bae H. (2019). Targeting of M2-like tumor-associated macrophages with a melittin-based pro-apoptotic peptide. J. Immunother. Cancer.

[24] Grivennikov S.I., Greten F.R., Karin M. (2010). Immunity, inflammation, and cancer. Cell.

[25] Roszer T. (2015). Understanding the Mysterious M2 Macrophage through Activation Markers and Effector Mechanisms. Mediat. Inflamm..

[26] Allavena P., Sica A., Solinas G., Porta C., Mantovani A. (2008). The inflammatory micro-environment in tumor progression: The role of tumor-associated macrophages. Crit. Rev. Oncol. Hematol..

[27] Martinez F.O., Gordon S. (2014). The M1 and M2 paradigm of macrophage activation: Time for reassessment. F1000Prime Rep..

[28] You Z., Shi X.-B., DuRaine G., Haudenschild D., Tepper C.G., Lo S.H., Gandour-Edwards R., White R.W.D.V., Reddi A.H. (2006). Interleukin-17 Receptor-Like Gene Is a Novel Antiapoptotic Gene Highly Expressed in Androgen-Independent Prostate Cancer. Cancer Res..

[29] Lan C., Huang X., Lin S., Huang H., Cai Q., Wan T., Lu J., Liu J. (2013). Expression of M2-Polarized Macrophages is Associated with Poor Prognosis for Advanced Epithelial Ovarian Cancer. Technol. Cancer Res. Treat..

[30] Xu J., Yu Y., He X., Niu N., Li X., Zhang R., Hu J., Ma J., Yu X., Sun Y. (2019). Tumor-associated macrophages induce invasion and poor prognosis in human gastric cancer in a cyclooxygenase-2/MMP9-dependent manner. Am. J. Transl. Res..

[31] Sumitomo R., Hirai T., Fujita M., Murakami H., Otake Y., Huang C.L. (2019). M2 tumor-associated macrophages promote tumor progression in non-small-cell lung cancer. Exp. Ther. Med..

[32] Tang X., Mo C., Wang Y., Wei D., Xiao H. (2013). Anti-tumour strategies aiming to target tumour-associated macrophages. Immunology.

[33] Ngambenjawong C., Gustafson H.H., Pun S.H. (2017). Progress in tumor-associated macrophage (TAM)-targeted therapeutics. Adv. Drug Deliv. Rev..

[34] Paull B.R., Yunginger J.W., Gleich G.J. (1977). Melittin: An allergen of honeybee venom. J. Allergy Clin. Immunol..

[35] Son D.J., Lee J.W., Lee Y.H., Song H.S., Kil Lee C., Hong J.T. (2007). Therapeutic application of anti-arthritis, pain-releasing, and anti-cancer effects of bee venom and its constituent compounds. Pharmacol. Ther..

[36] Jo M., Park M.H., Kollipara P.S., An B.J., Song H.S., Han S.B., Kim J.H., Song M.J., Hong J.T. (2012). Anti-cancer effect of bee venom toxin and melittin in ovarian cancer cells through induction of death receptors and inhibition of JAK2/STAT3 pathway. Toxicol. Appl. Pharmacol..

[37] Soman N.R., Baldwin S.L., Hu G., Marsh J.N., Lanza G.M., Heuser J.E., Arbeit J.M., Wickline S.A., Schlesinger P.H. (2009). Molecularly targeted nanocarriers deliver the cytolytic peptide melittin specifically to tumor cells in mice, reducing tumor growth. J. Clin. Investig..

[38] Lee C., Bae S.-J.S., Joo H., Bae H. (2017). Melittin suppresses tumor progression by regulating tumor-associated macrophages in a Lewis lung carcinoma mouse model. Oncotarget.

[39] Foillard S., Jin Z.-H., Garanger E., Boturyn D., Favrot M.-C., Coll J.-L., Dumy P. (2008). Synthesis and Biological Characterisation of Targeted Pro-Apoptotic Peptide. ChemBioChem.

[40] Javadpour M.M., Juban M.M., Lo W.-C.J., Bishop S.M., Alberty J.B., Cowell S.M., Becker C.L., McLaughlin M.L. (1996). De Novo Antimicrobial Peptides with Low Mammalian Cell Toxicity. J. Med. Chem..

[41] Kim H.Y., Kim S., Youn H., Chung J.-K., Shin D.H., Lee K. (2011). The cell penetrating ability of the proapoptotic peptide, KLAKLAKKLAKLAK fused to the N-terminal protein transduction domain of translationally controlled tumor protein, MIIYRDLISH. Biomaterials.

[42] Mai J.C., Mi Z., Kim S.H., Ng B., Robbins P.D. (2001). A proapoptotic peptide for the treatment of solid tumors. Cancer Res..

[43] Tian Y., Guo Y., Zhu P., Zhang D., Liu S., Tang M., Wang Y., Jin Z., Li D., Yan D. (2019). TRIM59 loss in M2 macrophages promotes melanoma migration and invasion by upregulating MMP-9 and Madcam1. Aging.

[44] Govindarajan B., Bai X., Cohen C., Zhong H., Kilroy S., Louis G., Moses M., Arbiser J.L. (2003). Malignant Transformation of Melanocytes to Melanoma by Constitutive Activation of Mitogen-activated Protein Kinase Kinase (MAPKK) Signaling. J. Biol. Chem..

[45] VValastyan S., Weinberg R.A. (2011). Tumor Metastasis: Molecular Insights and Evolving Paradigms. Cell.

[46] Noy R., Pollard J.W. (2014). Tumor-associated macrophages: From mechanisms to therapy. Immunity.

[47] Li Y., You M.J., Yang Y., Hu D., Tian C. (2020). The Role of Tumor-Associated Macrophages in Leukemia. Acta Haematol..

[48] Georgoudaki A.-M., Prokopec K.E., Boura V.F., Hellqvist E., Sohn S., Östling J., Dahan R., Harris R.A., Rantalainen M., Klevebring D. (2016). Reprogramming Tumor-Associated Macrophages by Antibody Targeting Inhibits Cancer Progression and Metastasis. Cell Rep..

[49] Arlauckas S.P., Garris C.S., Kohler R.H., Kitaoka M., Cuccarese M.F., Yang K.S., Miller M.A., Carlson J.C., Freeman G.J., Anthony R.M. (2017). In vivo imaging reveals a tumor-associated macrophage–mediated resistance pathway in anti–PD-1 therapy. Sci. Transl. Med..

